# Microbiology laboratory consolidation: Optimizing diagnostic efficiency and service quality—A case study from Dubai Health, United Arab Emirates

**DOI:** 10.1016/j.ijregi.2025.100779

**Published:** 2025-10-06

**Authors:** Rubina Lone, Adnan Alatoom, Anju Nabi, Bhavna Mathur, Maya Habou, Rubina Monga, Rupa Varghese, Sam Daniel, Shalinder Kour Hooda, Sujith Joseph, Hiba Saud Othman Alhumaidan

**Affiliations:** 1 Department of Pathology and Laboratory Medicine, Al Jalila Children's Specialty Hospital, Dubai, United Arab Emirates; 2 Department of Pathology and Laboratory Medicine, Latifa Hospital, Dubai, United Arab Emirates; 3 Department of Pathology and Laboratory Medicine, Dubai Hospital, Dubai, United Arab Emirates; 4 Department of Pathology and Laboratory Medicine, Rashid Hospital, Dubai, United Arab Emirates; 5 Department of Pathology and Laboratory Medicine, Hatta Hospital, Dubai, United Arab Emirates; 6 Department of Pathology and Laboratory Medicine, Dubai Health Headquarters, Dubai, United Arab Emirates

**Keywords:** Consolidation, Current Procedural Terminology, College of American Pathologists

## Abstract

**Objectives:**

This case study evaluated the 2024 consolidation of microbiology laboratory services within Dubai Health’s network, aiming to enhance diagnostic efficiency, reduce costs, and standardize service quality across tertiary hospitals, primary health care centers, and medical fitness centers.

**Methods:**

A hub-and-spoke model was implemented, with Al Jalila Children’s Specialty Hospital as the central hub for non-urgent testing and rapid response laboratories at peripheral sites for urgent testing. The phased approach included infrastructure upgrades, standardized protocols, optimized transport logistics, and a unified laboratory information system. Performance metrics included sample delivery rates, cost reductions, specimen rejection rates, proficiency testing, and test volume changes.

**Results:**

Consolidation achieved a 98.57% on-time delivery rate for routine samples and 97.90% for critical samples. Operational costs dropped by 6.1% for reagents, 62.7% for proficiency testing, and 19.01% for workforce, despite a 10.9% test volume increase. Specimen rejection rates decreased from 0.50% to 0.05%, and proficiency testing performance improved to 99.59%. The current procedural terminology code optimization boosted revenue.

**Conclusions:**

Dubai Health’s hub-and-spoke model enhanced efficiency, reduced costs, and standardized quality, offering a scalable framework for global health care systems. In addition to demonstrating enhanced efficiency and cost savings, this study provides insights from the Gulf region where published data remain limited. By outlining practical strategies, such as phased redeployment, robust transport logistics, and continuous clinical engagement, this work offers a scalable model adaptable not only to Gulf countries but also to diverse health care systems worldwide.

## Highlights

• Hub-and-spoke model centralized microbiology testing at Al Jalila Children’s Specialty Hospital.
• Unified protocols and electronic medical records system enhanced diagnostic consistency across sites.
• Optimized logistics ensured timely sample delivery and integrity.
• Workforce was optimized, cutting staff while boosting productivity.
• Current procedural terminology optimization and inventory control increased revenue and cut costs.

## Introduction

Clinical microbiology laboratories are essential for diagnosing infectious diseases, guiding antimicrobial treatment, and supporting public health [1]. However, fragmented operations across multiple sites often cause inefficiencies, duplicated efforts, and inconsistent service quality. Rising health care costs and shifting reimbursement models, such as the transition from fee-for-service to diagnosis-related group-based payments, pressure institutions to optimize operations while maintaining high-quality diagnostics [2,3]. One widely adopted strategy to address these pressures is laboratory consolidation by centralizing routine, high-volume, and non-urgent testing in a single location serving multiple institutions, which has been shown to improve efficiency, reduce costs, and enhance standardization [4]. Previous studies have highlighted several advantages of consolidation: decreased operational costs, enhanced diagnostic accuracy, broader test menu offerings, opportunity to synergize expertise, optimized resources, elevated standard of care for patients, methodological consistency across sites, and greater investment in staff development [[3], [4], [5]]. For example, a consolidated model implemented at Detroit Medical Center demonstrated a 13% gain in productivity and a 20% cost reduction per test compared with peer institutions [6]. To inform such initiatives, comprehensive workforce analyses, such as timed studies and Q-Probe studies aligned with College of American Pathologists (CAP) guidelines, are critical for identifying inefficiencies and optimizing resource allocation [7]. Similarly, risk assessments and quality improvement projects are essential to anticipate challenges, enabling proactive mitigation strategies and helping build laboratory capacities [8]. Financial analyses, including current procedural terminology (CPT) code optimization, further ensure accurate billing and revenue capture, enhancing the economic viability of consolidation efforts [9].

Despite these advantages, concerns about delayed turnaround times (TATs) or limited data access persist. Automation, optimized logistics, and robust information system integration can mitigate these risks, ensuring seamless service delivery [10,11]. Building on this approach, Dubai Health’s Department of Laboratory Medicine and Pathology has implemented a consolidated microbiology model to address similar challenges within its diverse health care network, incorporating timed and Q-Probe studies, risk assessments, and CPT optimization to guide the process [7,8].

Dubai Health’s diagnostic network supports tertiary care hospitals, primary health care centers (PHCs), and medical fitness centers (MFCs), serving varied clinical and population needs. Rashid Hospital, a 760-bed tertiary trauma center, specializes in neurosurgery, orthopedics, and intensive care. Al Jalila Children’s Specialty Hospital, with 200 beds, is the United Arab Emirates’ first dedicated pediatric tertiary hospital, offering advanced care in pediatric oncology, cardiology, and neurology. Dubai Hospital, with over 600 beds, manages chronic and complex diseases. Latifa Hospital, with 340 beds, focuses on high-risk obstetrics and neonatal care. Hatta Hospital provides general medical, surgical, and maternity services in the remote Hatta region. PHCs deliver community-based care, including chronic disease management and preventive screenings, whereas MFCs conduct high-volume health screenings for regulatory purposes.

Historically, Dubai’s public health care laboratories operated autonomously, leading to inconsistent service quality, duplicated efforts, and surplus capacity. Rising operational costs and inefficiencies, particularly, in resource-intensive microbiology, underscored the need for change. Microbiology’s high investment in biosafety infrastructure, specialized expertise, and advanced platforms, combined with low test volumes at individual hospitals, made it ideal for consolidation [4,12]. In early 2024, Dubai Health initiated a strategic review of laboratory operations, identifying microbiology as a high-impact area for transformation, and the microbiology service line was formally established, with a mandate to develop the transformative business plan. The Microbiology Service Expert Group included the following: Division Chief of Microbiology; consultant microbiologists; senior biomedical scientists; laboratory managers; infection prevention specialists; quality and patient safety coordinator; Information and Technology Coordinator; and representatives from pre-analytical, logistics, and revenue cycle management. Biomedical scientists/technologists were directly involved in study design and workflow mapping. Planning began in the first quartile of 2024, which proposed a consolidated service delivery model with a clear objective: to reduce operational and capital expenditures while enhancing service quality. The central tenet of this model was the consolidation of all non-urgent microbiology testing—defined as those with a TAT exceeding 2 hours—into a centralized, high-throughput reference laboratory. In addition, only STAT microbiology tests requiring rapid results would continue to be processed within hospital-based rapid response laboratories (RRLs). For clarity, “STAT” refers to microbiology testing requiring immediate processing included in Table 1.

**Table 1:** The core functions of the microbiology rapid response laboratories established at peripheral facilities as part of the consolidation initiative.

• Initial specimen accession for tracking purposes • Specimen quality assessment • Specimen rejection of inappropriately collected or poor-quality specimens • Specimen separation and aliquoting • Packaging and shipping of tests to CMS • Urinalysis • Cell counts and analysis of sterile fluids (i.e. cerebrospinal fluids) • Specimen tracking of overdue reports • Communication to the CMS

CMS, Central Microbiology Laboratory.

By mid-2024, formal approval was granted for the consolidation of microbiology services at Al Jalila Children’s Specialty Hospital, which was selected as the central hub due to its modern laboratory infrastructure and geographic accessibility. The phased transition of services commenced in August 2024, starting with low-volume tests and escalating to full-service centralization by October 2024. The consolidation aimed to improve operational efficiency, ensure uniform quality standards, reduce redundancy in equipment and staffing, and enhance the overall responsiveness of the microbiology service line across all Dubai Health facilities.

## Materials and methods

### Consolidation planning

Microbiology Service line expert group discussed a range of operating model options for the delivery of microbiology services across Dubai Health. These operational models include:

1. Status quo model: Continue independent laboratory operations.
  Advantages: Staff familiarity.
  Disadvantages: Persistent service variability, workforce shortages, limited technology investment, inability to achieve savings, and failure to meet consolidation mandates.
2. Intense collaborative working model: Harmonize methodologies without structural changes. Advantages: Staff familiarity, standardization.
  Disadvantages: Continued variability, workforce shortages, limited technological investment, and insufficient savings
3. Hub-and-spoke model: This model designated a central microbiology laboratory (CML) as the hub to handle high-volume and routine testing, whereas RRLs at other locations focused on STAT testing to deliver urgent results within 2 hours.
  Advantages: Standardization, equipment consolidation, staff efficiency, sustainable staffing, and advanced technology investment.
  Disadvantages: Requires site alterations, higher business continuity risks, staff impact, and uncertainty during implementation.

After studying the pros and cons of each of the models, options 1 and 2 were dismissed for failing to address inefficiencies or meeting cost-saving goals. The expert group decided to implement a hub-and-spoke model to consolidate microbiology services across its network. Al Jalila Hospital was chosen as the CML based on a rigorous evaluation of four key parameters: transport logistics, space availability, sample criticality, and sample volumes.

A detailed transport logistics analysis was conducted using retrospective data collected throughout 2023. This analysis tracked specimen transport times from the five tertiary care hospitals to a proposed central hub while also considering routes to PHCs and MFCs. The data set captured travel durations during peak and off-peak periods, sample volumes, specimen criticality, and spatial requirements for centralization, providing a holistic view of logistical demands.

Availability of space for centralization was another pivotal factor in selecting Al Jalila as the CML. The hospital offered substantial infrastructure, including dedicated areas for molecular diagnostics, bacteriology, and mycobacteriology labs, as well as storage for high-throughput equipment and reagents. This space accommodated the consolidation of services previously spread across multiple sites. Its existing pediatric-focused facilities also provided room for expansion, supporting the integration of advanced technologies such as automated culture systems and molecular platforms.

Criticality of samples played a significant role in the hub-and-spoke design. The CML’s central location minimized delays for these critical samples, with on-demand transport options ensuring delivery within 30 minutes when needed. In addition, RRLs at Rashid, Dubai, Latifa, and Hatta hospitals, as well as PHCs, were equipped to handle STAT testing with establishment of core functions at each of the RRLs sites (Table 1).

The pre-consolidation analysis of microbiology sample volumes processed at four Dubai Health hospitals was studied (Table 2). Al Jalila Hospital consistently reported the highest number of routine and specialized microbiology tests, including blood cultures, genital swabs, and respiratory specimens, indicating its robust testing capacity and supporting its designation as the central site for microbiology services post-consolidation.

**Table 2:** Pre-consolidation analysis of bacteriology sample volumes processed at four Dubai Health hospitals. This excludes tests including molecular microbiology, stool for ova and parasites, and infectious serology.

Detailed analysis	Al Jalila Children’s Specialty Hospital	Dubai Hospital	Hatta Hospital	Rashid Hospital
Anaerobic culture	32			
Biopsy culture: anaerobic and aerobic and gram stain	324	274	1	320
Blood culture routine	13395	8959	590	8736
Cath tip culture	56	59	1	89
Cerebrospinal fluid culture: anaerobic and aerobic and gram stain/cell count	484	141	5	586
Cystic fibrosis culture	26			
Ear culture: aerobic and gram stain	237	167	78	60
Eye culture: aerobic and gram stain	583	206	4	27
Fluid culture: anaerobic and aerobic and gram stain	322	581	14	801
Fungal micro and culture	78			500
Genital biopsy culture: anaerobic and aerobic and gram stain	0	48		
Genital fluid culture: anaerobic and aerobic and gram stain	0		1	
Genital swab culture: aerobic and gram stain	6832	3972	954	907
Mouth culture	2	4		
Methicillin-resistant *Staphylococcus aureus* culture	3222			
Nasal culture	12	61	17	8
Nasal scrapping for leprosy	0			9
Respiratory culture: aerobic and gram stain	1363	1451	71	2640
Semen culture: aerobic and gram stain	1	54		2
Stool culture: aerobic	1003	2723	295	1797
Throat culture	641	370	200	295
Umbilical culture: anaerobic and aerobic and gram stain	4	10	2	3
Urethral culture: aerobic and gram stain	2	66	2	49
Urine culture	21701	25837	4249	9274
Wound culture: anaerobic and aerobic and gram stain	1460	1730	226	3398
Total number of samples	51780	46173	6710	29501

### Implementation phases

Implementation of laboratory consolidation involves a structured, phased approach—beginning with comprehensive assessment and planning, followed by infrastructure upgrades, process standardization, workforce optimization, and culminating in centralized operations with continuous quality monitoring and stakeholder engagement. We used a four-phase implementation roadmap to guide the microbiology laboratory consolidation across Dubai Health in 2024 (Table 3).

**Table 3:** The four-phase implementation roadmap used to guide the microbiology laboratory consolidation across Dubai Health in 2024.

Phases	Description
Phase 1: Assessment and preparation (Q1 2024)	Workforce analysis: assessed staffing, workload, testing volumes, sample types, and TAT across sites. Monthly productivity studies to identify workflow improvements. Equipment and inventory mapping: condition, maintenance, and compatibility review. Data collation and analysis to identify trends and optimization opportunities.
Phase 2: Design and planning (Q2 2024)	Transport and financial assessments addressing logistical and budgetary challenges. Current procedural terminology optimization with revenue cycle management. Facility readiness assessment at Al Jalila, including infrastructure upgrades.
Phase 3: Implementation (Q3 2024)	Staff assessment: skill and staffing requirement evaluation. Standardization of standardized operating procedures for sample collection, processing, testing, and reporting. Creation of a directory of services detailing tests, TAT, and specimen needs. Formation of RRLs at low-volume sites for urgent testing. Staff training on new processes and technologies. Laboratory infrastructure optimization. Implementation of quality assurance measures.
Phase 4: Deployment and Evaluation (Q4 2024)	Execution of consolidation plan, including bacteriology centralization and RRL establishment. Continuous monitoring of Key Performance Indicators (KPIs)such as TAT, error rates, and customer satisfaction. Ongoing quality control and assurance. Continued staff training and development. Establishment of feedback channels for staff and stakeholders.

RRL, rapid response labs; TAT, turnaround times.

### Risk assessment

Before the centralization of microbiology services across Dubai Health’s facilities, a comprehensive risk assessment was undertaken to identify potential challenges that could affect the success of the restructuring initiative. The assessment was carried out through consultations with multidisciplinary teams including laboratory managers, clinicians, logistics personnel, and quality officers. Each risk was evaluated using a qualitative matrix that considered the potential impact on project outcomes and the likelihood of occurrence. This systematic approach enabled the team to prioritize risks and develop appropriate mitigation strategies. The assessment highlighted several key areas of concern, including staff turnover, pre-analytical vulnerabilities, sample loss during transport, increased TATs, and delays in sample transport affecting stability. Although staff turnover was considered a low-risk factor due to the presence of adequate staffing across sites, it was still recognized as a potential disruptor requiring monitoring. In contrast, pre-analytical errors and physician dissatisfaction were rated as medium-level risks, given the procedural changes and communication challenges introduced by the centralization. Loss of samples during inter-facility transport was assessed as having a high potential impact, despite a low probability of occurrence, because any such event could have significant implications for patient safety and institutional credibility. TAT increases were considered likely and potentially disruptive, especially in the early phases of implementation. Finally, delays in sample transport and the resulting threat to sample stability emerged as one of the most critical risks, with high likelihood and high potential impact due to the dependence on external courier logistics in the centralized model.

### Workforce analysis

In January 2024, a time-based workload study was conducted to optimize microbiology services across Dubai Health’s network. This study systematically measured the duration of all laboratory tasks, encompassing billable and non-billable tests, to evaluate resource allocation and identify areas of under- or over-productivity. Task durations were recorded to pinpoint inefficiencies and guide targeted interventions for workforce optimization during the centralization of microbiology services.

In addition, a Q-Probe study, aligned with the CAP guidelines, was performed to complement the time-based analysis [7]. Unlike the time-based study, which focused on task duration, the Q-Probe study calculated productivity by multiplying the number of billable tests by their volume, accounting for test complexity and quantity. Conducted as a third-party assessment by CAP, this study involved submitting data for external benchmarking against a global peer group. This dual approach provided a comprehensive assessment of laboratory performance, enabling the identification of suboptimal resource allocation and informing strategic interventions to enhance workflow efficiency.

### Financial analysis

To optimize financial performance, a centralized inventory system was implemented to track reagent and consumable use across microbiology networks to generate comprehensive consumption reports for transparency, streamline procurement, and minimize overstocking or shortages. Proficiency testing (PT) costs, including registration fees, testing materials, and shipping charges, were analyzed across all five hospitals to identify cost-saving opportunities. In addition, a comprehensive review of all microbiology tests to identify and correct incomplete or missing CPT codes was performed with the revenue cycle management team, ensuring accurate billing and revenue capture.

## Results

### Risk mitigations

To ensure the successful centralization of microbiology services, a comprehensive set of risk mitigation strategies was implemented to address findings from the risk assessment, promoting operational stability and clinical satisfaction. Staff retention was secured through proactive workforce planning, targeted engagement, and continuous training, minimizing turnover and ensuring expertise continuity despite the low risk. To counter the medium-level risks of pre-analytical variability and physician dissatisfaction, standardized sample collection protocols were deployed across referring sites, complemented by clinical team education and formal communication channels to maintain engagement and address feedback promptly [13]. The unlikely risk of sample loss was mitigated by enhancing the transport chain with real-time tracking and chain-of-custody verification, improving accountability and traceability, further described in the Transport logistic section. To manage potential increases in TATs due to higher sample volumes, preemptive optimizations, such as workload balancing, auto-validation of results, and real-time dashboards with process audits, were introduced, stabilizing operations after minor initial delays. Sample integrity during transport was preserved through a dedicated courier service with high frequency, scheduled routes, ensuring delivery within pre-analytical windows to maintain diagnostic accuracy [8]. In addition, the availability of six board-certified pathologists on 24/7 rotating schedules, including on-call and weekend duties, enhanced diagnostic quality, particularly, for complex cases such as pediatric infections across regional referral laboratories [14]. These targeted strategies collectively enabled the centralization of microbiology services with minimal compromise to quality, efficiency, or clinical satisfaction, ensuring a robust and sustainable diagnostic framework.

### Transport logistics

Sample transport to the central laboratory was optimized by scheduling specimen transport every 2 hours, 24/7, across peak and off-peak periods, covering all hospital, PHC, and MFC routes to Al Jalila Hospital. This 2-hour interval ensured the stability of non-transport medium specimens because delays beyond this could degrade sample quality, particularly, for cultures and molecular assays. For critical samples such as blood cultures, an on-demand transport protocol enabled dispatch within 30 minutes of request, even outside scheduled intervals, addressing urgent clinical demands. Initial challenges included peak-hour delays, with Dubai hospital to Al Jalila Hospital occasionally reaching 64 minutes due to traffic. These were mitigated by optimizing routes, such as adding a secondary pathway via Rashid Hospital, and increasing van frequency during peak hours. Post-implementation, the transport system achieved a 97% on-time delivery rate for non-critical samples, which arrived at CML within the 2-hour target, in time for processing without compromising quality. This logistics framework not only ensured timely specimen transport but also reduced pre-analytical errors, contributing to the overall success of the microbiology consolidation.

### Standardization and quality

Policies and procedures were harmonized across all sites to ensure consistent diagnostic protocols [12]. Around 230 standardized operating procedures were standardized across reporting locations. Standardized operating procedures were developed for sample collection, processing, and testing and result reporting, reducing variability in results and improving quality by minimizing errors, Unification of reporting standardized data formats and terminology across the network. All labs adopted a single laboratory information system with a comprehensive microbiology module, ensuring uniform result reporting. This enhanced quality by reducing misinterpretation risks and facilitating communication with clinicians.

Standardization of equipment improved quality by ensuring consistent performance across all sites. The CML and RRLs adopted uniform platforms for molecular diagnostics and automated systems, resulting in reduced inter-lab variability in test results and increased savings from bulk purchasing of reagents. Removing redundant equipment further enhanced savings and quality. Duplicate analyzers and outdated machines were phased out, consolidating resources at the CML and RRLs. This eliminated maintenance costs for redundant units while improving quality by focusing on well-maintained, high-performance devices.

TAT and other key performance indicators were optimized to enhance quality and efficiency. The CML handled routine tests with a TAT of 98.57%, meeting the ≥95% target, whereas RRLs delivered STAT results within defined time, achieving 97.90% TAT for urgent cases. This consistency improved patient outcomes by ensuring timely diagnoses, especially for critical infections requiring rapid intervention. The specimen rejection rate dropped to 0.05% against a benchmark of 0.50%. Evaluation of CAP PT performance increased to 99.59% against a benchmark of 90% [6].

### Workforce optimization and productivity

#### Time-based workload study

The analysis revealed inefficiencies, identifying an excess of 25 full-time equivalents (FTEs) among the 121 employees, indicating underproductive areas. Conversely, overproductive hospitals highlighted the need for additional FTEs in specific segments.

This detailed mapping pinpointed suboptimal resource allocation within microbiology services, enabling targeted interventions. By reallocating staff to overproductive departments and management roles during the centralization process, the workforce was streamlined to 98 staff by October 2024. This restructuring enhanced productivity, optimized workflow processes, and maintained efficiency, ensuring high-quality microbiology service delivery across Dubai Health’s facilities.

#### Benchmark productivity such as Q-probe study

The results of the Q-Probe study corroborated findings from the timed study, reaffirming areas of under productivity within the microbiology division. By leveraging insights from the timed study and the Q-Probe analysis, we were able to implement strategic initiatives aimed at enhancing productivity, improving TATs, and, ultimately, delivering superior microbiology services to our patients and health care providers. The Q-Probe study done by College of American Pathologist showed a productivity increase from 15% pre-consolidation to 73% post-consolidation.

Despite a 10.9% increase in test volume, this restructuring cleared 26 FTEs, resulting in a potential 19.01% reduction in workforce while maintaining efficiency and ensuring high-quality microbiology service delivery across Dubai Health’s facilities.

#### Financial performance

Operational expenses for reagents and consumables decreased by 6.1%, from $21,755,861 in 2023 to $20,428,885 in 2024. The centralized inventory system reduced reagent-related errors by 9.9% and achieved 10% cost savings through bulk ordering and reduced stockouts, despite a 10.9% increase in test volume. Consolidation of PT activities reduced costs by 62.7%, from $215,322 to $81,316, while improving quality and consistency. The CPT code review identified incomplete or missing codes for 27 bacteriology, 16 serology, and nine molecular tests, increasing the total from 62 to 144 CPT codes. This optimization generated an additional $5,555,041 in revenue, enhancing financial performance across the microbiology network.

Compared with models in Europe, North America, and Australasia, the Dubai Health centralization differed in its context of centralized government oversight, phased workforce redeployment, and strong emphasis on specialist retention. Gulf geography and climate also shape transport logistics, with the remotest referring to hospital located approximately 132.5 km from the central hub. Although no category 4/Biosafety Level 4 (BSL 4) facilities were involved, the consolidation emphasized biosafety 2 and 3 practices appropriate for routine diagnostics. The lessons learned, including the importance of preserving specialist expertise, standardizing pre-analytical protocols, and creating a robust and reliable transport system, are broadly applicable to other health care systems.

#### Post-consolidation outcomes

Table 4 summarizes the key outcome areas assessed after the consolidation of microbiology services at Dubai Health in 2024. Each domain reflects specific improvements observed at the CML and RRLs across the network. The indicators include quantitative metrics and qualitative improvements.

**Table 4:** Overall effectiveness and sustainability of the consolidation initiative.

Outcome area	Description
Operational optimization	Streamlined workflows and automation minimized turnaround time and enhanced efficiency, at the central microbiology laboratory processing 4,390,741 tests (this includes all microbiology tests) in 2024, reducing manual handling by 20%.
Quality assurance	Regular audits and College of American Pathologists proficiency testing-maintained accuracy and compliance, achieving 99.73% performance and reducing errors by 5%.
Staff development	Ongoing training ensured proficiency in new technologies and protocols, with 100% staff participation, improving skill retention by 15%.
Cost optimization	Continuous monitoring identified savings through contract renegotiation (8% IT cost reduction) and inventory management 6.1% reagent savings even though the volumes increased by 10%).
Adaptation to emerging needs	Agile responses to changing testing demands (e.g., Mpox) and regulations ensured compliance and rapid deployment of new assays.
Continuous improvement culture	Encouraged feedback, innovation and collaboration, reducing process inefficiencies by 10%.
Market evaluation and service offering	Explore external client opportunities (e.g., private clinics) with targeted marketing, potentially adding 5% to revenue
Training for external candidates	Initiated programs for medical technologists and technicians, enhancing skills and positioning Dubai Health as a laboratory education leader.
Automation solutions	Future investment in automation systems (e.g., sample sorting robots) to streamline processing and result reporting, with potential to improving efficiency by 25% and accuracy by 5%.

## Discussion

The ongoing shift from fee-for-service to value-based and capitated reimbursement models has significantly transformed the operational landscape of health care systems, including clinical laboratories. As Dranove et al. [15] observed, the rise of managed care arrangements has redefined laboratories from revenue-generating units to cost centers, placing a premium on efficiency and cost containment. Within this context, the consolidation of diagnostic services—including high-complexity disciplines, such as microbiology—emerges not only as a strategic financial response but also as a mechanism for enhancing operational performance.

Centralization offers distinct advantages: economies of scale, elimination of redundant infrastructure, and standardized processes across health care networks [5]. These are particularly impactful in microbiology, where the complexity and resource demands of testing necessitate robust, high-throughput, and standardized workflows [4]. Dubai Health’s experience aligns with these broader global trends, underscoring that thoughtful consolidation can be a sustainable strategy to maintain quality while controlling costs.

Gray et al. [16] have highlighted the benefits and challenges of microbiology laboratory consolidation, reporting improved TATs, reduced operational costs, expanded test menus, and centralized expertise as key advantages. However, the authors also identified potential risks, such as sample loss during transport, service disruption due to rapid staff downsizing, and decreased diagnostic accuracy [16]. At Dubai Health, we addressed these risks through a phased implementation strategy, the retention of specialized microbiology staff, and the establishment of a dedicated specimen transport system—measures that allowed us to preserve diagnostic integrity and minimize disruptions across the network.

Carter et al. [9] documented a 5-year consolidation of three microbiology laboratories at the University of South Alabama Medical Center, achieving improved TATs, elimination of four full-time positions, and annual net savings of $103,000 after accounting for courier costs. Although financial savings were not the primary driver for Dubai Health, similar outcomes were observed. Efficiency and TAT improved notably, without substantial staff reductions. Instead, we prioritized optimization of workflows, maintenance of specialized expertise, and implementation of a dedicated courier system akin to the model described by Carter et al., ensuring timely and secure specimen delivery [9].

The consolidation at Dubai Health also facilitated improved revenue capture through CPT code optimization and reduction in operational costs, despite an overall increase in test volumes. Challenges encountered included initial overstaffing, resolved via strategic reassignments; productivity variability, mitigated through removal of manual work [11]; and billing discrepancies, addressed collaboratively with revenue cycle management teams. Although the short evaluation period limits assessment of long-term outcomes, the early results suggest measurable benefits. Future directions include continued workforce adjustments, expansion of diagnostic offerings, and further automation investments [11].

Robinson et al. [13] observed that traditional microbiology models—characterized by proximity and frequent interaction between laboratory staff and clinical teams—are increasingly being replaced by centralized laboratory structures, potentially eroding the vital communication necessary for optimal infectious disease management. Similarly, Carter et al. [9] cautioned that an exclusive financial focus in consolidation may inadvertently impair communication between microbiologists and health care providers. Although electronic health records enhance data access, they do not replicate the nuance of direct consultation.

In addition to demonstrating enhanced efficiency and cost savings, this study provides evidence from the Gulf region, where published data remain limited. By outlining practical strategies, this work offers a scalable model adaptable not only to Gulf countries but also to diverse health care systems worldwide.

Peterson et al. [14] expanded on these concerns, highlighting the risks of delayed specimen processing, prolonged turnaround for critical tests such as Gram stains and antimicrobial susceptibility profiles, and the potential for specimen degradation during transport. They also warned of a declining role for clinical microbiologists in educating future clinicians—a consequence of consolidation and evolving medical curricula. These factors contribute to complex challenges in delivering high-quality infectious disease diagnostics across diverse care settings.

Our experience at Dubai Health reflects many of these challenges, particularly, about maintaining effective communication between microbiologists and clinicians and ensuring timely sample processing despite expanded transport distances. However, through rigorous planning, dedicated logistics infrastructure, and enhanced consultative services, we have successfully mitigated these risks. As a result, we preserved diagnostic quality and clinical collaboration throughout the consolidation process.

## Conclusion

This study describes the successful implementation of microbiology laboratory consolidation within the Dubai Health network, resulting in enhanced diagnostic efficiency, improved financial performance, and greater public health responsiveness. By leveraging careful planning, stakeholder engagement, and dedicated transport and consultative infrastructure, Dubai Health offers a viable and scalable model for health care systems worldwide seeking to adapt to the evolving economic and clinical demands of modern laboratory medicine.

## Declaration of competing interest

The authors have no competing interests to declare.
